# THE DAILY LIFE OF HEALTHY AGING: CONSIDERING PERCEPTIONS, APPRAISALS, AND CONSEQUENCES

**DOI:** 10.1093/geroni/igac059.477

**Published:** 2022-12-20

**Authors:** Patrick Hill

## Abstract

For decades, researchers have shown how individuals deal with both stressful and positive events is central to our understanding of healthy aging. Multiple studies have demonstrated that reactivity to daily events shapes our health and wellbeing, with the potential for compounding effects to cause greater hindrance by older adulthood. This literature also notes that individuals differ significantly in their appraisals, resources, and experience with stressors, leading to the need for multimethod research into the role that daily events play in adult development. The current series of talks will investigate these topics using a variety of methodologies, while considering multiple perspectives on the study of stress and wellbeing. First, Megan Wilson will consider the deleterious outcomes associated with a specific form of daily stressors, everyday experiences of discrimination, focusing on how daily discrimination may negatively impact adults’ sense of purpose in life. Second, Eric Cerino will discuss a collaborative effort that compares approaches to studying control beliefs at the dispositional and daily level, showing that researchers may benefit from considering daily stressor control as a construct unique from general beliefs. Third, Jessica Maras will also consider the role of perceptions, by examining how cognitive appraisals of stressful events impact future health outcomes. Fourth, Patrick Klaiber will present meta-analytic work that combines daily studies to consider whether the benefits of positive events on wellbeing differ by age. Taken together, these studies provide new insights into the daily life of healthy aging, and how it differs based on individual differences and stressor type.

